# P-1886. Utilizing Artificial Intelligence to Predict Triage in Pediatric Outpatients with Prolonged and Recurrent Fever

**DOI:** 10.1093/ofid/ofae631.2047

**Published:** 2025-01-29

**Authors:** Guyu Li, Nan Huo, Amir B Orandi, Elizabeth H Ristagno

**Affiliations:** Mayo Clinic, Rochester, Minnesota; Mayo Clinic, Rochester, Minnesota; Mayo Clinic, Rochester, Minnesota; Mayo Clinic, Rochester, Minnesota

## Abstract

**Background:**

Prolonged and recurrent fevers in pediatric outpatients often require specialist consultations, presenting a challenge in accurately triaging to the correct pediatric subspecialty. This study aims to develop an artificial intelligence (AI)-based model to accurately direct pediatric patients to appropriate subspecialties.

**Table 1.**
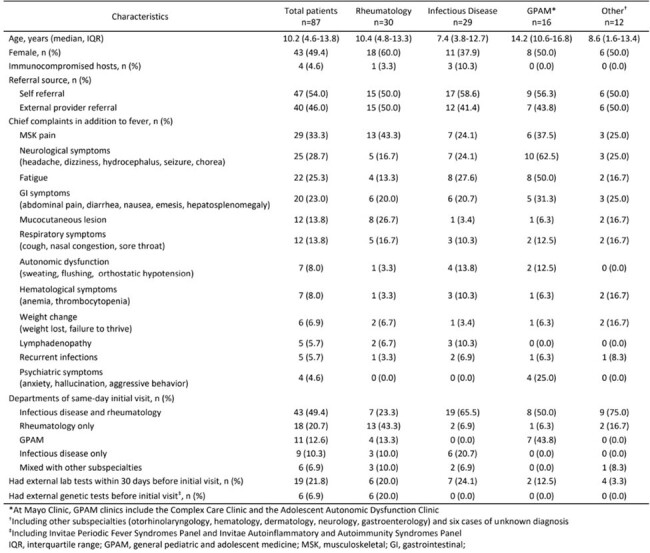
Patient characteristics and the department confirming the final diagnosis

Table 1. Patient characteristics and the department confirming the final diagnosis

**Methods:**

A retrospective study was conducted at the Mayo Clinic, Rochester, from December 1, 2020, to January 1, 2023, involving pediatric patients aged 0 to 18 referred for recurrent fever or fever of unknown origin. Six machine learning models, including gradient boosting, random forest, support vector machines (SVM), k-Nearest Neighbor (KNN), logistic regression (LR) and decision tree, were developed using 7 variables (demographics, chief complaints, referral sources, departments of same-day initial visit, and lab tests results within 30 days before initial visits). Data were extracted from external electronic health records (EHR) using Mayo Data Explorer and Lab Information Management Systems. Precision, Recall, F1 Score, and area under the curve (AUROC) were selected to measure the performance.

**Table 2.**
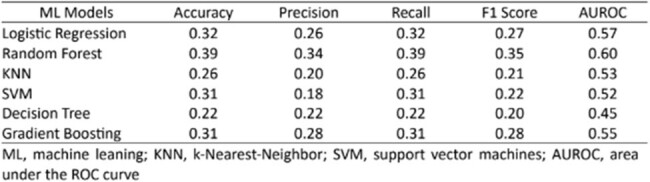
Performance of six machine learning models

Table 2. Performance of six machine learning models

**Results:**

The study evaluated 87 pediatric patients with a median age of 10.2 years (IQR: 4.6-13.8), including 43 (49%) females (Table 1). External provider referrals were noted in 40 (46%) patients. Musculoskeletal pain was the most common chief complaint, identified in 29 (33%) patients, in addition to prolonged or recurrent fever. Infectious disease and rheumatology divisions jointly saw 43 (49%) patients during the initial visit. Sub-optimal performance was observed in six machine learning models (Table 2). The random forest model showed the best results (Accuracy=0.39, Precision=0.34, Recall=0.39, F1 Score=0.35, AUROC=0.60) (Figure).

**Figure.**
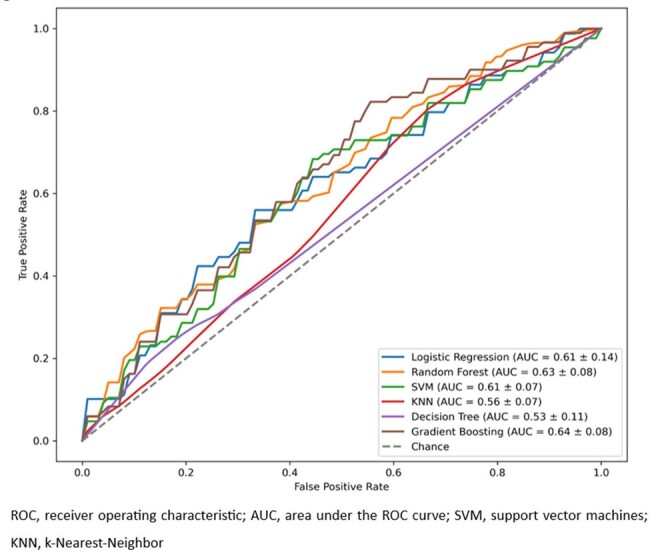
ROC curves for various models

Figure. ROC curves for various models

**Conclusion:**

This study demonstrates the potential of AI-based models for outpatient triage prediction in pediatric patients with recurrent fever, using EHR data to optimize health resource utilization. This data serves as the groundwork to expand and refine this AI triage tool.

**Disclosures:**

All Authors: No reported disclosures

